# The Multicultural Personality Questionnaire (SF-40): Adaptation and Validation of the Spanish Version

**DOI:** 10.3390/ijerph18052426

**Published:** 2021-03-02

**Authors:** Lynn Patricia Summerfield, Vicente Prado-Gascó, María del Carmen Giménez-Espert, Patricia Mesa-Gresa

**Affiliations:** 1 Social Sciences Department, European University of Valencia, Paseo de la Alameda, 7, 46010 Valencia, Spain; lynn.summerfield@universidadeuropea.es; 2 Social Psychology Department, Faculty of Psychology, University of Valencia, Avda. Blasco Ibañez 21, 46010 Valencia, Spain; vicente.prado@uv.es; 3 Nursing Department, Faculty of Nursing and Chiropody, University of Valencia, C/Jaume Roig s/n, 46010 Valencia, Spain; 4 Psychobiology Department, Faculty of Psychology, University of Valencia, Avda. Blasco Ibañez 21, 46010 Valencia, Spain; Patricia.Mesa@uv.es

**Keywords:** multiculturalism, personality, university students, validation

## Abstract

The coexistence of diverse cultures in our society indicates the need to examine the factors related to the success of multicultural interactions. The study aims were to examine the psychometric characteristics of the Spanish version of the Multicultural Personality Questionnaire short form (MPQ-SF40), in a convenience sample of 392 university students. Then, the effect of sex and age was assessed, and finally, the levels and percentiles of multicultural personality were measured. The scale’s validity was assessed with exploratory and confirmatory factor analysis (EFA and CFA). Reliability was evaluated with Cronbach’s alpha, composite reliability (CR), and average variance extracted (AVE). The final structure of the MPQ-SF40 consisted of 18 items grouped into five factors that present adequate psychometric properties. Sex-specific differences in MPQ-SF40 were statistically significant for factor 1, cultural empathy, and for factor 5, flexibility; women showed greater values. When age was analyzed, significant low correlations were obtained. The students showed medium to high levels of multicultural personality. The highest levels correspond to the personality factors of cultural empathy and open-mindedness. The use of the Spanish version of the MPQ-SF40 seems justified to determine students’ multicultural personality traits, developing intervention programs to improve social support and the interpersonal relations between students.

## 1. Introduction

Multiculturalism, arising as a consequence of international mobility, is a fundamental part of contemporary life. As a result, individuals find themselves mixing with people from diverse cultures in both personal and professional spheres [1]. Their ability to succeed in such diversity is a key aspect to an individual’s personal, social, and professional adjustment [2]. Consequently, successful multicultural interaction can lead to increased levels of well-being; perceived social support; and in relation to the academic and professional sphere, better performance [1] through enabling greater efficiency in problem solving and higher levels of creativity, among other advantages.

As a reaction to growing diversity in the field of higher education, universities are integrating a more international dimension to their curricula on an ever-growing scale, in many cases as part of the process of internationalization they are immerse in. In addition, as some authors highlight, other consequences of the increasing levels of multiculturalism could be the need for graduates who are capable of interacting in an effective and productive manner within a culturally diverse workforce [3]. In the same way, other research analyzes the intercultural competences students need to acquire to subsequently work in intercultural contexts. In this respect, a consideration of interpersonal aspects, such as attitudes towards culturally diverse groups or multiculturalism, is also gaining relevance [4]. However, in higher education, some issues surrounding multiculturalism have been identified as a potential source of tension or causing a barrier due to concepts such as prejudice. This manifests itself in many students rejecting interaction with international students, preferring to work in a familiar cultural environment [5]. Therefore, some authors highlight the importance of addressing the problems and complexities related to the interaction between different nationalities on an international campus [6].

To this end, research is required to analyze the concept of success in multicultural interaction [2], with a multicultural attitude being identified as an important predictor of such success [4,7,8]. As sustained by the Theory of Planned Behaviour (TPB), attitudes can be affected by the individual’s internal and external conditions. In the case of multiculturalism, research has traditionally focused more on external, contextual, or social factors rather than interpersonal aspects, although the latter seem especially useful since they are much more stable than other types of contextual variables. Among the internal variables posed by the TPB, personality traits stand out [4,9]. Some studies emphasize the impact that personality has on such intercultural interactions, labelling it as a key aspect to take into consideration [9]. Thus, when dealing with a multicultural environment, more relevance could be given to narrow matrix personality models that set out more specific traits. Trait analysis models already exist in the field of personality studies which are widely accepted and implemented, such as the Big Five [10]. The model’s name refers to five broad personality factors: extraversion (how active, social, and expressive an individual is), neuroticism (the degree to which an individual displays negative affect), agreeableness (how interpersonally friendly and sensitive an individual is towards others), conscientiousness (the degree to which an individual completes tasks as best as possible in the absence of external motivation), and openness (an individual’s propensity for curiosity and inventiveness). These broad personality traits have a moderate impact on human behavior and are reproduced in all cultures [11]. However, the Big Five do not provide the level of specificity required to understand how personality can affect aspects such as specific attitudes and intentions, job performance and satisfaction, or multicultural success [12]. Conversely, narrow traits have a more profound impact on an individual’s behavior [13] and are better predictors of behavior than the broad traits in socio-cultural contexts [14,15]. Multicultural Personality theory proposes that narrow personality traits exist that can predict the variation of multicultural competence (adaptability and cultural adjustment, intercultural comfort, and effectiveness in intercultural environments). The most frequently cited theory of multicultural personality is that proposed by Van der Zee and Van Oudenhoven [14], which is based on the concept of “multicultural efficiency”.

Hence, the researchers Van der Zee and Van Oudenhoven [14] defined multicultural personality as, “success in the fields of professional effectiveness, personal adjustment and intercultural interactions” (p. 293). This model combines narrow personality traits that appear to be more relevant in gauging the performance of an individual in a multicultural context [9,16]. Since its conception, the Multicultural Personality model has been implemented in different studies, obtaining acceptable results in research on satisfaction with the workplace abroad [17], effectiveness in intercultural mentoring [18], student adaptation to exchange programs [19], and multicultural effectiveness in university students [20]. The authors developed the Multicultural Personality Questionnaire (MPQ) [16] with the aim of focusing on those factors that contribute to an individual’s success in a culturally diverse environment, on intercultural effectiveness and adaptation [16,21]. There are numerous scales to measure similar aspects of an individuals’ attitude to or level of multiculturalism, such as the Munroe Multicultural Attitude Scale Questionnaire (MASQUE) [22], whose psychometric properties are discussed below; the Multicultural Personality Inventory (MPI) [23], which is a long instrument of 70 items that risks respondent fatigue; the Multicultural Personality Inventory short form (MPI-SF) [24], in which the validation study results should be considered as preliminary until multiple replications can be completed.

Hence, the MPQ was developed as a more precise alternative to general personality scales. It has received wide empirical support internationally as one of the most robust questionnaires for assessing multicultural personality and intercultural competence in general [23,25]. It was initially created to evaluate the intercultural effectiveness of international students and expatriates during their stay abroad but has currently been used in different populations and contexts [26]. There is evidence of a positive relationship between MPQ and productivity [27]. Other studies link MPQ dimensions to many intercultural outcomes, such as cultural integration [28] and cultural identity at work [29]. The MPQ also predicts attitudes such as international students’ adjustment [30] and intercultural effectiveness [26].

In addition, the shorter version of the scale, the Multicultural Personality Questionnaire short form (MPQ-SF40) [16] has been validated in diverse populations, mainly focusing on educational environments or college and university students [31,32,33] or adult workers and their environment [34] on an international scale in Europe, Asia and North America with around 40 empirical studies [25], reporting adequate psychometric properties. The questionnaire is easy to implement and provides an adequate evaluation of an individual’s openness to multicultural contexts. However, to date, it appears not to have been adapted and tested in Spain. Diverse authors have highlighted the need to consider such precise models of personality in certain contexts, specifically, a model that accounts for those personality traits that could be considered useful in the process of adaptation of an individual to a culturally diverse environment [16,21], precisely, the environment one can increasingly find on increasingly international university campuses. In the same way, research has explored numerous factors that could influence student adjustment to college life, such as family environment [35], satisfaction in relationships, and conflict management [36]. However, one field that has received comparatively less research is that of personality. The main aim of this research, therefore, is to adapt and validate the MPQ-SF40 in Spanish university students to fill an existing gap in this field. Additionally, the effect of sex and age was assessed, and finally, the levels and percentiles of multicultural personality were measured.

## 2. Materials and Methods

A cross-sectional study was carried out in a convenience sample of 392 undergraduates from two Spanish Universities located in the Valencian Community. The inclusion criteria were students who voluntarily agreed to participate, unpaid, and signed the informed consent. Participants were from all degree years and of diverse nationalities. All participants were informed of the objectives of the study, the confidentiality of the data, and that non-participation would not have any consequence on their academic development. This study was authorized by the Human Research Ethics Committee of the University of Valencia H20190312094249.

### 2.1. Instrument

Multicultural Personality Questionnaire–SF40: The short form (MPQ-SF40) [16] from the original Multicultural Personality Questionnaire was used, which was composed of 91 items (MPQ) [20]. An adaptation to Spanish of the forty-item scale evaluates five factors: cultural empathy (8 items assess the ability to empathize with culturally diverse individuals, e.g., sets others at ease), social initiative (8 items measure actively approaching social situations and taking the initiative, e.g., takes the lead), open-mindedness (8 items assess the absence of rigid prejudices towards diverse cultural groups, e.g., tries out various approaches), emotional stability (8 items measure the ability to remain calm and collected under stressful conditions, e.g., is nervous), and flexibility (8 items assess an attitude of seeing new situations as positive challenges and adaptation to the new cultural environment, e.g., has fixed habits). A Likert scale of five points was used from I completely identify with (5) to I don’t identify at all with (1). Higher scores indicate a higher level of each factor, except in the case of flexibility, where higher scores indicate less flexibility. The scale displays adequate psychometric properties in previous studies (α = 0.72 to 0.82) [12], (α = 0.71 to 0.85) [37] and in this study (α = 0.60 to 0.80). In the current study, the reliability of the MPQ-SF40 scale is acceptable (α = 0.80).

### 2.2. Procedure

The self-report questionnaires were completed in the classroom by the participants, which lasted around 20 min. The data collection phase was conducted between November 2015 and February 2016. The international methodological standards for language adaptation of an instrument established by the International Test Commission (ITC, 2001) were taken into account in the Spanish adaptation of the Multicultural Personality Questionnaire-SF40 [38].

### 2.3. Statistical Analyses

SPSS software (version 22), EQS software (Structural Equation Modelling Software, Version 6.2) [39] and FACTOR software (Rovira i Virgili University, Tarragona, Spain) [40] were used to carry out the statistical analyses of this study. First, descriptive analysis of every item was calculated. The validity of the scale and its reliability was also analyzed, using the following measures: Cronbach’s alpha, composite reliability (CR), and average variance extracted (AVE). The exploratory factor analysis (EFA) and confirmatory factor analysis (CFA) were carried out. Then, the effect of sex and age was measured, and finally, the levels and percentiles of multicultural personality were calculated.

## 3. Results

### 3.1. Sample Characteristics

A sample of 392 undergraduates from two Spanish Universities located in the Valencian community using convenience sampling, with ages from 18 to 41 years old, mean age 21.96 years (*SD =* 3.49), participated in this study. With regard to sex, 50.40% were men, while 49.40% were women. With regard to the degree year, percentages of participants in each year were: first, 21.90%; second, 26.90%; third, 24.80%; fourth, 25.30%; and fifth, 1.10%. In terms of the nationality, 47.60% were Spanish, 29.20% Italian, 6.20% English, 5.70% German, 2.70% French, and the remaining 8.60% were students of other nationalities. The average score obtained in their studies was, with merit 43.10%, followed by good 39.90%, pass 11.90%, and outstanding 5%. Finally, regarding proficiency in English, nearly half of the respondents, 48%, stated that they had an intermediate level, 29.40% indicated an advanced level, while for 13.10%, it was their mother tongue, and 9.50% had a basic level.

### 3.2. Item Analysis

When the descriptive analysis of the 40 items that compose the MPQ-SF40 was performed, it was observed that the data set was similar to a normal distribution, based on the indexes of skewness and kurtosis (see Table 1), so that the use of maximum likelihood factor tests was allowed. Table 1 also shows the results of the homogeneity indexes or the item-total correlation of the items. In the majority of the items, the corrected item-total correlation reached values higher than 0.40. It was also observed that all the items of the questionnaire contributed to increasing the internal consistency of the scale, since their elimination decreased the Cronbach’s alpha value. Regarding the values of skewness and kurtosis, the items showed a range of ±1.

The reliability of the MPQ-SF40 scale is acceptable (α = 0.80) [41], with Cronbach’s alphas that vary from 0.60 (open-mindedness) to 0.80 (social initiative) for each factor.

### 3.3. Factor Analysis

First, the suitability of the sample was assessed using the Kaiser–Meyer–Olkin (KMO) and Bartlett’s sphericity tests [42,43]. KMO analyzed an optimal value (0.81), and Bartlett’s sphericity test results (χ^2^ = 3067.70; df = 378; *p* ≤ 0.001) were also adequate. Subsequently, to perform the EFA, given that skewness and kurtosis values lower than −2 or 2 were observed, it was decided to follow the process recommended by Lloret-Segura, Ferreres-Traver, Hernández-Baeza, and Tomás-Marco [44] and use the maximum likelihood method. To determine the number of common factors in which the items are grouped, parallel analysis (PA) was used. PA selects those components that have common factors presenting eigenvalues greater than those that would be obtained by chance [45]. This technique was developed to be used on the original correlation matrix. However, its use has also been recommended to identify the number of common factors [46]. First, following the recommendation of Lloret-Segura et al. [44], normalized direct Oblimin rotation was used. Notwithstanding, the analysis was repeated using the normalized Varimax rotation, since the correlations between the factors established by the EFA were quite low (<0.30) when applying the Oblimin rotation model, not allowing us to ensure the relationship between the factors proposed. The EFA was carried out using the FACTOR program [40] with the 40 items of the MPQ-SF40 questionnaire, which recommended the grouping of the items into five common factors. Then, the application of the EFA was fixed to five factors, and after elimination of items with reduced factor loadings (<0.40), the scale was reduced to 28 items. The fit of this solution was adequate with the root mean square root of the residuals (RMCR) value of 0.38 (<0.0584, the expected RMCR value for an acceptable model) [40] and a goodness of fit index (GFI) of 0.98 (>0.95) [47]. The variance explained by the three factors was 49.76%.

After the EFA was carried out following the recommendations suggested by the parallel analysis method, several CFAs were performed with the robust Satorra–Bentler correction [48] in order to verify the factor structure obtained by the EFA (28 items grouped into five dimensions) and, on the other hand, to contrast the fit of the original proposal by Van der Zee et al. [16]. The different models proposed were analyzed, taking into account both the correlation between pairs of factors and the absence of correlation. The indicators of fit of the various proposed models are shown in Table 2.

The estimation of the different models was performed using the robust maximum likelihood estimation method to control for the possible absence of multivariate normality. As can be seen in Table 2, the models with the best fit are those in which factors are correlated. After several specifications of the initial models, the two proposals (the one coming from the EFA and the one composed of the five original factors of the instrument) showed a good fit. In these two proposals, a similar number of items were eliminated, 21 items in the one derived from the EFA and 22 in the original proposal. The proposal derived from the EFA presents a more difficult theoretical interpretation when compared with the original, since some factors include items from two dimensions of the original grouping. For this reason, it was decided to discard this model and use the one composed of 18 items encompassed in the five dimensions of the original instrument (final instrument Table A1 English version and Table A2 Spanish version, Appendix A). This model presents adequate fit (S-B χ^2^ = 213.89; df = 125; *p* <0.05; χ^2^/df = 1.91; non normed fit index (NNFI) = 0.90; comparative fit index (CFI) = 0.92; incremental fit index (IFI) = 0.92; and root mean square error of approximation (RMSEA)= 0.046) [49,50,51] (see Table 2).

With respect to the reliability of the scale (see Table 3), it should be noted that the reliability of the AVE was higher than the correlation between pairs of factors. The CR of the MPQ-SF40 scale ranged from 0.55 to 0.81. As for AVE indicators, they show values higher than the cut-off point (>0.50) recommended by Fornell and Larker [52], except in the factors of cultural empathy, open-mindedness and emotional stability.

### 3.4. Validity of the Scale

To be able to increase the empirical evidence of construct validity, the convergent and discriminant validity of the scale was calculated. Convergent validity appeared adequate, as the scale items significantly correlated with the latent variables. The T values for the variables oscillated from 4.73 to 13.71 (t > 1.96) and were significant at the 0.05 level. The loadings of each average factor were higher in some measures (>0.70) (Table 4) [53].

On the other hand, discriminant validity was evaluated by means of the AVE test. The correlations between the various factors were less than 0.85 [54], as seen in Table 4. Moreover, the square root of the AVE was verified to be greater than the correlation between pairs of factors [52], complying with this criterion for all dimensions.

### 3.5. Sensitivity of the Scale to Sex and Age Effects

With the aim of obtaining the effects of age and sex on the differences in MPQ-SF40, different analyses were carried out. The sex-specific mean scores were measured by the T test, Cohen’s d, and effect-size r. Then, the correlation between the different dimensions, also including age, was tested according to sex (Table 5). Finally, the factorial invariance regarding sex was tested. The sex-specific differences were statistically significant for factor 1, cultural empathy (*T* = −5.08; women: *M* = 4.04, *SD* = 0.57; men: *M* = 3.74, *SD* = 0.61; *p* = <0.001; effect size with *Cohen’s d* = 0.51, *r* = 0.25), and for factor 5, flexibility (*T* = −2.31; women: *M* = 3.04, *SD* = 0.90; men: *M* = 2.83, *SD* = 0.85; *p* = 0.022; effect size with *Cohen’s d* = 0.24, *r* = 0.12). Generally, women showed greater values than men.

When age was analyzed, significant low negative correlations (*p* < 0.01) were obtained with flexibility (*r* = −0.15, *p* = 0.04) in the case of men, and low and positive correlation with open-mindedness (*r* = 0.15, *p* = 0.04) in the case of women. In general, higher values were also observed in the rest of the correlations in women compared to men. Finally, Satorra–Bentler scaled chi square statistics using SBDIFF [55,56] were used to assess the factorial invariance. The results of S–B Scaled Difference = 10.88, df = 12; *p* = 0.54 shows equal form invariance and equal factor loading invariance (if item 11 and 40 are removed).

### 3.6. Levels and Percentiles of Multicultural Personality

The students showed medium to high levels of multicultural personality. The highest scores correspond to the personality factors of cultural empathy (*M* = 3.89; *SD* = 0.60) and open-mindedness (*M* = 3.64; *SD* = 0.56). Conversely, the flexibility factor showed the lowest score (*M* = 3.06; *SD* = 0.88). Finally, so as to facilitate the understanding of the data, the percentiles from 10 to 90 were obtained considering the whole sample and regarding sex (Table 6).

## 4. Discussion

Given the undeniable presence of multiculturalism in today’s societies, the importance of achieving appropriate multicultural interaction and the role that personality factors play in being successful are gaining relevance. The internationalization of the workplace is increasingly evident, and as a result, employees are required who know how to perform within that context [57,58]. Universities are shaping their curricula with an international focus to meet the demands of the labor market, pleasing their domestic students while attracting increasing numbers of international students [20,59]. Within the framework of higher education, it is considered fundamental to integrate an intercultural or international dimension in education as part of the internationalization process taking place in many universities [60], and this dimension should include the development of intercultural competencies in students [61]. However, despite the importance of multiculturalism in society as well as in an educational context, several studies note apathy and refusal to interact with international peers by students [31,62]. Therefore, research needs to be developed that addresses the analysis of success in multicultural interactions [2,63]. While numerous studies have focused on contextual or social aspects of the multicultural group (roles, performance, and challenges), which seem to affect success in intergroup relationships [64], as established by Kimmel and Volet [65], there seems to be hardly any studies that focus on individual aspects, such as personality.

Therefore, the main aim of the present study was to adapt the MPQ-SF40 [16] to the Spanish context. The importance of this personality scale lies in the fact that it has been specifically designed to determine an individual’s disposition for achieving success in an international context and predict multicultural effectiveness [16,20], in contrast to other more general personality scales, and thus satisfies the need for such a questionnaire. Moreover, this instrument allows rapid assessment, so it can be very useful in a myriad of research scenarios. Various studies have evaluated the validity of MPQ-SF40 in differing samples of students from different countries [19], not having been validated in Spain. The final version of the scale consisted of 18 items distributed among five dimensions. This model presents a significant chi-square (S-Bχ2 = 213.89; df = 125; *p* < 0.05) and a value of the normed chi-square (χ2/df = 1.91), which indicated a good fit, as it shows a value of less than five [50]. The RMSEA showed a value of 0.046, which meets the minimum acceptable fit criteria (equal to or less than 0.08) [51]. Likewise, the other indices showed a good fit of the model, as they showed values higher than 0.90: NNFI = 0.90, CFI = 0.92, and IFI = 0.92 [49]. In line with other studies, such as Korol’s [37] in a sample of university students in Portugal, CFA confirmed the five-factor structure, goodness of-fit indexes values of 0.90, and RMSEA = 0.076. The final version of the scale appears to be a promising self-report measure to predict multicultural effectiveness in a Spanish context. However, this study provides initial evidence, and new replications could be completed in more students from Spanish universities.

The scale evaluates five different aspects of personality, cultural empathy, social initiative, open-mindedness, emotional stability, and flexibility, and is, therefore, considered a more accurate alternative in the more specific field of multicultural environments compared to the broader and more general personality scales [16]. Compared to broader personality factors, and the Big Five model in particular, the construction of the multicultural personality has demonstrated a higher predictive validity in the context of intercultural interaction [12,19]. Moreover, it is among the most robust questionnaires for assessing multicultural personality as well as intercultural competence in general [23,25]. Furthermore, van Oudenhoven and van der Zee [32] reported that a multicultural personality was relevant for both individuals who have moved abroad as well as for individuals living in culturally diverse communities.

The sex-specific differences were statistically significant for factor 1, cultural empathy, and for factor 5, flexibility, in women. In the present study, when analyzing age, significant low negative correlations were obtained with flexibility in the case of men, and low and positive correlation with open-mindedness in the case of women. The findings on the sex of participants were in line with other studies, showing that women tend to be more empathetic towards different cultures and in their cultural perceptions [66,67]. Other studies by Van Der Zee and Van Oudenhoven [68] found that sex influenced four dimensions of MPQ (social initiative, open-mindedness, emotional stability, and flexibility), yet age seemed unrelated. These different results may indicate that further research is needed to clarify the effects of age and sex on the differences in the dimensions of the MPQ-SF40.

With regard to determining the multicultural personality levels in Spanish university students, the present study showed medium to high levels [16,19], as well as providing percentiles to interpret the results of the MPQ-SF40. The highest scores corresponded to the personality factors of cultural empathy and open-mindedness, while students showed to identify less with the aspects related to flexibility [32,37]. The results suggest that most students are open to multicultural contexts. It should be noted that the cultural empathy dimension has been established as one of the most frequently mediated variables in both cultural effectiveness and intercultural competence [37]. It is defined as the ability to easily identify with individuals from other cultures [37]. In the open-mindedness dimension, the findings showed one of the highest average scores and is considered an important component in counteracting prejudice and fostering favorable attitudes towards individuals from a different culture [25]. Therefore, the data obtained are relevant if we wish to determine the personality traits that can predict positive attitudes towards culturally diverse groups.

Moreover, the present study could have a number of practical implications for professionals within an international campus university context. This group of professionals could extend to program designers and staff, as well as faculty. For instance, by taking into account the levels of multicultural personality among students, an intervention program could be introduced to raise awareness and tolerance of cross-cultural differences. These interventions could adopt both a formal and informal approach, as formal international emphasis in the curricula would have increased effectiveness if supported at the same time by informal actions [69]. Buddy systems could be encouraged, for instance, so that both national and international students work together to help each other in all aspects of university life, helping to breakdown the ingroup–outgroup categorization, and giving students opportunities to establish contact with each other.

In spite of the advantages of this study, it is necessary to point out the limitations, such as the non-probabilistic sampling methodology used, the limited sample size, and geographical location, as well as the removal of several items due to very reduced factor loadings, which make its generalization difficult. It would, therefore, be interesting to conduct future research on a larger scale, improving item factor loadings and maintaining more items, with the inclusion of other regions of Spain, using probability sampling, in addition to conducting comparisons of the two groups of students from different universities and degree programs. Future studies should also expand the research to other Spanish-speaking countries, as well as other samples of the population where analysis of the multicultural personality could be interesting, such as multinational companies or in the political field, as well as conducting comparisons between the different characteristics of the samples. Despite these limitations, the adaptation of the MPQ-SF40 to the Spanish context shows initial evidence regarding the assessment of multicultural personality traits of Spanish university students. Additionally, it provides information on their potential success and ease of adaptation to a multicultural environment, such as in the students’ possible future workplace. Thus, it offers the possibility of its implementation as a diagnostic tool in planning an intervention in this context.

## 5. Conclusions

Multiculturalism is a fundamental part of contemporary life and in the field of higher education, since universities are integrating an increasingly international dimension. A key aspect that determines the effectiveness of intercultural interactions is multicultural personality traits. The present research provides initial findings to suggest that it has the potential to be a useful clinical and research measure instrument able to evaluate students’ multicultural personality traits in a Spanish context; identify educational needs; and develop intervention programs in order to improve social support, the interpersonal relations of the students, as well as their academic performance. Finally, the percentiles provide an easy interpretation of the scores and allow a comparison with other student samples.

## Figures and Tables

**Table 1:** Analysis of the Multicultural Personality Questionnaire–SF40.

Complete Questionnaire (α = 0.80)	*M*	*SD*	*r_jx_*	*α-x*	*Skewness*	*Kurtosis*
Cultural Empathy: α = 0.77						
MPQSF1	4.03	0.79	0.47	0.75	−0.55	0.22
MPQSF16	3.82	0.83	0.40	0.76	−0.37	0.04
MPQSF18	3.87	0.88	0.46	0.75	−0.59	0.21
MPQSF23	3.93	0.86	0.59	0.73	−0.62	0.09
MPQSF27	4.03	0.86	0.51	0.74	−0.74	0.26
MPQSF32	3.80	0.96	0.44	0.75	−0.48	−0.23
MPQSF36	3.93	0.80	0.48	0.75	−0.59	0.57
MPQSF37	3.94	0.75	0.44	0.75	−0.52	0.35
Social Initiative: α = 0.80						
MPQSF8	3.35	1.01	0.51	0.78	−0.40	−0.09
MPQSF10	3.63	0.93	0.55	0.77	−0.62	−0.22
MPQSF29	3.86	0.99	0.55	0.77	−0.44	−0.47
MPQSF33	3.84	0.95	0.44	0.78	−0.22	−0.55
MPQSF7	3.60	0.95	0.57	0.77	−0.22	−0.35
MPQSF3	4.04	1.03	0.53	0.77	−0.83	−0.15
MPQSF4	3.35	1.20	0.47	0.78	−0.27	−0.82
MPQSF22	3.49	1.03	0.46	0.78	−0.31	−0.34
Open-Mindedness: α = 0.60						
MPQSF2	3.71	0.75	0.39	0.54	−0.03	−0.44
MPQSF9	3.80	0.83	0.43	0.53	−0.48	0.04
MPQSF12	3.66	0.87	0.31	0.56	−0.26	−0.16
MPQSF13	4.02	0.87	0.34	0.55	−0.65	0.11
MPQSF15	3.55	0.96	0.15	0.61	−0.34	−0.32
MPQSF19	3.37	1.07	0.25	0.58	−0.13	−0.62
MPQSF30	3.95	0.89	0.41	0.53	−0.49	−0.22
MPQSF40	3.14	0.96	0.17	0.61	0.05	−0.21
Emotional Stability: α = 0.63						
MPQSF11	2.61	1.11	0.28	0.61	0.28	−0.51
MPQSF34	2.86	1.10	0.21	0.63	0.02	−0.73
MPQSF16	2.48	1.16	0.41	0.58	0.49	−0.49
MPQSF20	3.09	1.10	0.27	0.62	0.03	−0.65
MPQSF21	2.53	1.09	0.37	0.59	0.33	−0.51
MPQSF25	3.03	1.22	0.50	0.55	−0.07	−0.86
MPQSF31	2.44	1.20	0.25	0.62	0.48	−0.74
MPQSF38	3.62	1.03	0.33	0.60	−0.53	−0.20
Flexibility: α = 0.74						
MPQSF5	2.93	1.15	0.45	0.71	0.04	−0.64
MPQSF14	3.04	0.96	0.49	0.70	0.02	−0.24
MPQSF17	3.70	1.06	0.33	0.73	−0.47	−0.51
MPQSF24	2.83	1.12	0.54	0.69	0.00	−0.63
MPQSF26	3.70	0.96	0.34	0.73	0.30	−0.46
MPQSF28	3.64	0.87	0.40	0.72	−0.42	0.20
MPQSF35	3.02	0.99	0.49	0.70	0.03	−0.38
MPQSF39	3.31	0.96	0.43	0.71	−0.22	−0.16

Note: M = mean; SD = standard deviation; r_jx_ = item-total correlation; α-x = Cronbach’s alpha if it eliminates the element; MPQSF = Multicultural Personality Questionnaire Short Form.

**Table 2:** Scale goodness of fit indices MPQ-SF40.

Initial Model		S-B χ^2^ (df)	χ^2^ (df)	χ^2^/*df*	RMSEA (CI)	CFI	NNFI	IFI
(1) EFA 28 items and 5 factors.	5 factors, 28 items (correlated)	794.22 (340)	891.82 (340)	2.62	0.063 (0.057–0.069)	0.79	0.77	0.80
	5 factors, 28 items (not correlated)	903.10 (350)	1012.21 (350)	2.89	0.069 (0.063–0.074)	0.75	0.73	0.75
	5 factors, 19 items (correlated)	243.48 (142)	272.01 (142)	1.92	0.046 (0.036–0.055)	0.92	0.91	0.93
	5 factors, 19 items (not correlated)	346.33 (152)	387.49 (152)	2.55	0.061 (0.052–0.069)	0.86	0.84	0.86
(2) Original proposal Van der Zee et al. [16] composed of 40 items and 5 factors.	5 factors, 40 items (correlated)	1840.57 (730)	2095.96 (730)	2.87	0.068 (0.064–0.072)	0.61	0.59	0.62
	5 factors, 40 items (not correlated)	2018.46 (740)	2290.75 (740)	3.10	0.073 (0.069–0.077)	0.55	0.53	0.56
	5 factors, 18 items (correlated)	213.89 (125)	238.44 (125)	1.91	0.046 (0.035–0.056)	0.92	0.90	0.92
	5 factors, 18 items (not correlated)	363.50 (135)	406.34 (135)	3.01	0.070 (0.61–0.80)	0.80	0.77	0.80

Note: S–B = Satorra–Bentler; df = degrees of freedom; RMSEA = root mean square error of approximation (≤0.08); CI = RMSEA confidence interval; CFI = comparative fit index; NNFI = non normed fit index; IFI = incremental fit index; CFI, NNFI, IFI (≥0.90); χ^2^/df (≤3.00).

**Table 3:** Results of the confirmatory factor analysis with factor loadings, Cronbach’s alpha, composite reliability and average variance extracted for the MPQ-SF40.

Items		Factor Loading	CI of Cronbach’s Alpha	CR	AVE
	Factor 1 Cultural Empathy		0.71 (0.66–0.75)	0.71	0.34
MPQSF6		0.431			
MPQSF18		0.566			
MPQSF23		0.715			
MPQSF27		0.646			
MPQSF32		0.508			
	Factor 2 Social Initiative		0.79 (0.75–0.82)	0.81	0.59
MPQSF7		0.718			
MPQSF8		0.843			
MPQSF33		0.731			
	Factor 3 Open-Mindedness		0.54 (0.46–0.61)	0.55	0.24
MPQSF2		0.476			
MPQSF9		0.621			
MPQSF30		0.443			
MPQSF40		0.401			
	Factor 4 Emotional Stability		0.57 (0.49–0.64)	0.58	0.32
MPQSF11		0.481			
MPQSF21		0.649			
MPQSF25		0.545			
	Factor 5 Flexibility		0.74 (0.69–0.78)	0.76	0.52
MPQSF5		0.663			
MPQSF14		0.529			
MPQSF24		0.921			

Note: CR = composite reliability; AVE = average variance extracted; CI = confidence interval of Cronbach’s alpha.

**Table 4:** Interfactorial correlation matrix and the AVE values of the MPQ-SF40.

	F1	F2	F3	F4	F5
Factor 1—Cultural Empathy	0.58				
Factor 2—Social Initiative	0.12 **	0.77			
Factor 3—Open-Mindedness	0.28 **	0.55 **	0.49		
Factor 4—Emotional Stability	0.22 **	0.00	0.03	0.56	
Factor 5—Flexibility	−0.03	0.10	0.07	0.11 *	0.72

* p < 0.05, ** *p* < 0.01. Note: the square root of the AVE for each factor is in the diagonal of the correlation matrix.

**Table 5:** Pearson correlations according to sex between the factors of the MPQ-SF40 scale and the age variable.

	1		2		3		4		5	
	M	F	M	F	M	F	M	F	M	F
1.										
2.	−0.04	0.01								
3.	0.02	0.04	0.26 **	0.10						
4.	0.05	0.15 *	0.36 **	0.29 **	0.58 **	0.55 **				
5.	−0.08	0.14	0.06	−0.08	0.14	0.23 **	0.18 *	0.21 **		
6.	−0.15 *	0.02	0.04	−0.03	−0.03	−0.12	0.00	−0.13	0.05	0.04

** p < 0.01; * *p* < 0.05. Note: 1 = age; 2 = cultural empathy; 3 = social initiative; 4 = open-mindedness; 5 = emotional stability; 6 = flexibility; M = male; F = female.

**Table 6:** Percentiles for interpreting MPQ-SF40.

	Total					Women					Men				
	F1	F2	F3	F4	F5	F1	F2	F3	F4	F5	F1	F2	F3	F4	F5
10	3	2.67	3	2.33	1.67	3.25	2.33	2.75	2.33	1.67	3	2.67	3	2.27	1.67
20	3.40	3	3.25	2.43	2	3.60	2.67	3.25	2.67	2.33	3.20	3	3.15	2.33	2
30	3.60	3	3.25	2.67	2.67	3.80	3	3.25	2.67	2.67	3.40	3	3.25	2.67	2.33
40	3.80	3.33	3.50	2.67	2.67	3.92	3.33	3.50	3	2.67	3.60	3.33	3.50	2.67	2.67
50	4	3.67	3.50	3	3	4	3.59	3.50	3	3	3.80	3.67	3.75	3	3
60	4	3.67	3.75	3	3.33	4.20	3.67	3.75	3	3.33	4	3.67	3.75	3	3
70	4.20	4	4	3.33	3.33	4.40	4	4	3.33	3.67	4	4	4	3.33	3.33
80	4.40	4.33	4.25	3.33	3.67	4.60	4	4	3.67	3.67	4.20	4.33	4.25	3.33	3.67
90	4.80	4.67	4.50	3.67	4	4.80	4.37	4.25	3.67	4.33	4.60	5	4.50	3.67	4

F1 = cultural empathy; F2 = social initiative; F3 = open-mindedness; F4 = emotional stability; F5 = flexibility.

## Data Availability

The data presented in this study are available on request from the corresponding author.

## Appendix A

**Table A1:** Multicultural Personality Questionnaire (MPQ-SF40) English version.

**Cultural Empathy**
…Sets others at ease.
…Enjoys listening to others.
…Pays attention to the emotion of the others.
... Is a good listener.
…Enjoys getting to know others profoundly.
**Social Initiative**
…Takes the lead.
…Is often the driving force behind things.
…Takes initiative.
**Open-Mindedness**
…Tries out various approaches.
…Is looking for new ways to attain his/her goal.
…Has a broad range of interests.
…Is a trendsetter in societal developments.
**Emotional Stability**
…Keeps calm when things don´t go well.
…Gets upset easily.
…Is nervous.
**Flexibility**
…Likes routine.
…Has fixed habits.
…Looks for regularity in life.

**Table A2:** Multicultural Personality Questionnaire (MPQ-SF40) Spanish version.

**Empatía** **Cultural**
…hace que los demás se sientan cómodos.
…disfruta escuchando a los demás.
…presta atención a las emociones de los demás.
...sabe escuchar a los demás.
…disfruta conociendo en profundidad a la gente.
**Iniciativa** **Social**
…asume el mando.
…suele tener iniciativa.
…toma la iniciativa.
**Mente** **Abierta**
…experimenta con diferentes enfoques.
…busca nuevas formas de lograr sus objetivos.
…tiene una amplia variedad de intereses.
…marca tendencia
**Estabilidad** **Emocional**
…mantiene la calma cuando las cosas no van bien.
…se enfada fácilmente.
…es nerviosa.
**Flexibilidad**
…aprecia la rutina.
…es de hábitos fijos.
…busca la rutina en su vida.
